# Editorial: Neural responses for rehabilitation of the elderly: evidence from the micro, meso to macro scale

**DOI:** 10.3389/fnagi.2023.1208578

**Published:** 2023-05-15

**Authors:** Le Li, Sheng Li, Howe Liu, Wenxin Niu, Chuhuai Wang

**Affiliations:** ^1^Institute of Medical Research, Northwestern Polytechnical University, Xi'an, China; ^2^McGovern Medical School, University of Texas Health Science Center at Houston, Houston, TX, United States; ^3^Department of Physical Therapy, Allen College, Waterloo, IA, United States; ^4^School of Medicine, Shanghai Yangzhi Rehabilitation Hospital, Tongji University, Shanghai, China; ^5^Department of Rehabilitation Medicine, First Affiliated Hospital, Sun Yat-sen University, Guangzhou, China

**Keywords:** rehabilitation, elderly, stroke, Parkinson's disease, motor function, neuromodulation

The 19 articles in the Research Topic “*Neural Responses for Rehabilitation of the Elderly: Evidence from the Micro, Meso, to Macro Scale*” cover a variety of neural responses after motor exercise and rehabilitation intervention in older adults as well as in people with neurological disease. Among them, three original research articles addressed the impact of noninvasive brain stimulation, such as repetitive transcranial magnetic stimulation (rTMS) and continuous and intermittent theta burst stimulation (iTBS), on the functions of post-stroke and Parkinson's disease (PD). One review article examined the effect of rTMS on Alzheimer's disease and another provided a summary of non-invasive brain stimulation (NIBS) for osteoarthritis (OA). Moreover, four other articles highlight the effects of exercise therapy, such as Tai Chi, on mild cognitive impairment in the elderly, dance movement therapy (DMT) for neurodegenerative diseases, exercise intervention on social distancing in middle-aged and elderly patients with chronic low back pain, as well as robotic-assisted rehabilitation treatment of hand function. Further, nine articles covered various topics related to applied basic research on the elderly, including four articles on functional near-infrared spectroscopy (fNIRS) for subacute stroke, age-related differences in response to visual stimuli from EEG, the relationship between motor imagery (MI) and age-related fatigue, and post-stroke sensory deficiency in response to cold stimulation from EEG as well as five articles focused on the peripheral neuromuscular system, such as dual-task gait analysis in middle-aged to older people, the role of the diaphragm in postural stability and visceral function in PD, H-Reflex from lateral gastrocnemius among elderly people with peripheral neuropathy, and sarcopenia related to body composition and gait analysis. Last but not least, one basic research article covered the association between mitochondrial function and rehabilitation of PD by exosomal mRNA and lncRNA expression profiles.

## Exercise interventions

Tai Chi is a form of light-to-moderate-intensity mind-body exercise for seniors. In a review, Wang, Liu et al. showed that Tai Chi can improve cognitive functions and alleviate the accompanying symptoms of mild cognitive impairment in the elderly by potentially activating the expression of signals in different brain regions, altering their neural connectivity, increasing the brain volume, and modulating brain-derived neurotropic and inflammation factors. However, more studies are required to determine the frequency and duration of training to optimize the beneficial effects of Tai Chi on mild cognitive impairment.

DMT is the psychotherapeutic use of movement as a process that promotes the emotional, social, cognitive, and physical integration of the individual. In a systematic review, Wu et al. discussed the effects of DMT on motor function, cognitive deficit, mood, and quality of life in people with neurodegenerative diseases. This review showed that DMT can effectively improve motor function and cognitive deficits in neurodegenerative diseases. Future research on the effects of DMT on Alzheimer's disease requires scientific design, large sample size, long-term comprehensive intervention, and clear reporting standards. The same team also investigated the effect of exercise on social distancing in middle-aged and elderly patients with chronic low back pain. The authors concluded that the 8-week exercise intervention cannot only shorten the social distance and improve the abnormal behavior of social distancing regulation of middle-aged and elderly patients with chronic low back pain, but can also relieve pain, disability, and negative emotions. More studies may be needed to explore the long-term effects of exercise intervention, and further work is required to determine whether this new finding equally applies to other types of exercise.

Li, Zhang et al. implemented a hybrid brain computer interface (BCI) paradigm based on MI and steady-state visual evoked potential (SSVEP). In this study, EEG data from 12 healthy participants were collected, and the activation regions of THE MI-SSVEP paradigm were identified by power spectral density. This study verified the clinical effect of THE MI-SSVEP intervention paradigm for 61 stroke patients by applying robot assisted therapy with the MI-SSVEP intervention paradigm, which demonstrated significant functional improvement in these patients.

## Non-invasive brain stimulation

In various neurological diseases, NIBS has been actively explored. It is believed that NIBS induces excitatory changes in the underlying cerebral cortex in a non-invasive manner and lasting changes in neuroplasticity. Cao et al. conducted a randomized, case-control study of rTMS combined with respiratory muscle training for pulmonary rehabilitation after ischemic stroke. The authors found that the combined intervention showed a stronger increase in lung function detection indexes than respiratory muscle training alone and that the combined intervention could improve lung functionality after acute ischemic stroke. As a special kind of rTMS, iTBS has been proved to recover the function balance in stroke patients when conducted on the cerebellar vermis (Wang, Huang et al.). Zheng,Chen et al. designed and proposed a study protocol on cerebellar continuous theta burst stimulation (cTBS) for aphasia rehabilitation, which may further help researchers to investigate the efficacy of cTBS treatment on the right cerebellum in augmenting language recovery in chronic post-stroke aphasia. In order to analyze the effects of rTMS on global cognitive function in Alzheimer's disease, Zhang, Sui et al. conducted a systematic review and meta-analysis. The authors found that rTMS is a safe, potentially effective treatment and can induce long-lasting effects for cognitive impairment in AD. Another review from Zhu et al. summarized the therapeutic effects and mechanisms of different NIBS techniques (including TMS, electrical stimulation, ultrasound stimulation, and random noise stimulation) in OA, and clarified the potential of NIBS as a treatment choice for OA, which may provide prospects and suggestions for further research.

## Signal processing in aging or related disease: from central nervous system to peripheral neuromuscular system

Li, Chen et al. investigated EEG characteristics during MI by comparing young and elderly participants, and studied convolutional neural networks (CNN) classification for the elderly population in terms of fatigue analysis in both frontal and parietal regions. The authors demonstrated that the elderly are less affected by the level of cognitive fatigue during MI compared to the young subjects as the controls. The deep learning method also provides a potentially feasible option for the application of MI-BCI in the elderly by considering ERD and fatigue. Zhang, Jiang et al. also analyzed age-related differences in the transient EEG response in visual BCIs from visual evoked potential (VEP)/motion onset VEP (mVEP) and SSVEP/SSMVEP between the younger group and the elderly group. Their findings showed that the amplitudes of P1 elicited by motion onset are significantly higher in the senior group, which might be a potential advantage for seniors if mVEP-based BCIs is used. EEG could also be applied to study reduced elementary somatosensation after stroke. Huang et al. designed a new configuration for the measurement of post-stroke elementary thermal sensation by non-painful cold stimulation and the post-stroke cortical responses were investigated and compared with unimpaired persons. The results revealed that post-stroke cortical responses during stimulation and sensory deficiency were characterized by a wide distribution of representative EEG relative spectral power bands, lowered resolution toward different temperatures, and extensive activated sensory cortical areas. As well as EEG, fNIRS was also proved to be a useful tool to investigate reorganization of the brain network after stroke. Yuan et al. analyzed hemispheric dominance differences in the task-state motor network properties of subacute stroke by fNIRS and the results revealed that the changes in macroscale cortical network indicators were similar between the left hemisphere stroke (LHS) and the right hemisphere stroke (RHS) groups, while those of the mesoscale indicators were different. The authors revealed that the brain network characteristics of RHS were affected by the severity of the dysfunction.

Meanwhile, quantitative evaluations of peripheral neuromuscular systems also attract researchers' interests. Zheng, Lang et al. applied dual-task, three-dimensional gait analysis to mild cognitive impairment and age-matched controls, and principal component analysis was conducted to select the key biomechanical indexes from spatial-temporal parameters. Their results showed that there were significant differences in dual-task cost cadence during walking calculation tasks between the two groups, which may prove the potential value for application in the early identification of mild cognitive impairment in the clinic. Age-induced sarcopenia may negatively affect walking stability and increases the risk of falls, which represents the leading cause of accidental death in the elderly. Fan et al. analyzed and contrasted body the composition and gait characteristics of those with sarcopenia in relation to health controls. In the study, significant differences were found in certain gait parameters between elderly with sarcopenia and normal elderly participants, which were related to absolute muscle strength, suggesting that sarcopenia was a disease mainly caused by decreased muscle mass.

In an opinion article, Chen et al. reviewed the development of the motor unit number estimation (MUNE) method and discussed the feasibility and challenges of simulation-free MUNE, which is important to study motor units related to basic organizational and functional elements of neuromuscular control. The hoffmann reflex (H-reflex) assesses peripheral neuropathy (PN) adaptation in aged people, and Song et al. tried to find a reliable muscle among triceps surae during standing and walking in the PN population. Their results showed that LG was more reliable than SO and MG.

In patients with PD, Yu et al. found that gender and Hoehn and Yahr stages play significant roles in diaphragm thickness and exertion during resting breathing. The diaphragm's functionality is significantly correlated with the patient's postural stability, vocal capability, as well as with the respiratory, gastrointestinal, and urological functions. From the same lab, a randomized controlled trial study conducted on two small groups (intervention vs. control) compared how exosomal mRNA and lncRNA expression profiles may change after a 2-week rehabilitation intervention. The study revealed that the exosomal mRNA can be significantly upregulated while the lncRNAs can be significantly downregulated after the 2-week rehabilitation in comparison with the control group or in pre- and post-comparison within the PD patients; however, how the diaphragm might response to the rehabilitation was not assessed. The mRNA is related to mitochondrial respiration and lncRNA to pathogenesis of the disease. This may provide micro-level evidence why rehabilitation is beneficial to patients with PD.

## Author contributions

All authors listed have made a substantial, direct, and intellectual contribution to the work and approved it for publication.

